# Sensitivity to the two peptide bacteriocin plantaricin EF is dependent on CorC*, *a membrane‐bound, magnesium/cobalt efflux protein

**DOI:** 10.1002/mbo3.827

**Published:** 2019-03-19

**Authors:** Dustin D. Heeney, Vladimir Yarov‐Yarovoy, Maria L. Marco

**Affiliations:** ^1^ Department of Food Science & Technology University of California‐Davis Davis California; ^2^ Department of Physiology and Membrane Biology University of California‐Davis Davis California

**Keywords:** antimicrobial resistance, bacteriocins, lactic acid bacteria, *Lactobacillus*, probiotics

## Abstract

Lactic acid bacteria produce a variety of antimicrobial peptides known as bacteriocins. Most bacteriocins are understood to kill sensitive bacteria through receptor‐mediated disruptions. Here, we report on the identification of the *Lactobacillus plantarum *plantaricin EF (PlnEF) receptor. Spontaneous PlnEF‐resistant mutants of the PlnEF‐indicator strain *L. plantarum *NCIMB 700965 (LP965) were isolated and confirmed to maintain cellular ATP levels in the presence of PlnEF. Genome comparisons resulted in the identification of a single mutated gene annotated as the membrane‐bound, magnesium/cobalt efflux protein CorC. All isolates contained a valine (V) at position 334 instead of a glycine (G) in a cysteine‐β‐synthase domain at the C‐terminal region of CorC. In silico template‐based modeling of this domain indicated that the mutation resides in a loop between two β‐strands. The relationship between PlnEF, CorC, and metal homeostasis was supported by the finding that PlnEF‐resistance was lost when PlnEF was applied together with high concentrations of Mg^2+^, Co^2+^, Zn^2+^, or Cu^2+^. Lastly, PlnEF sensitivity was increased upon heterologous expression of LP965 *corC* but not the G334V CorC mutant in the PlnEF‐resistant strain *Lactobacillus casei *BL23. These results show that PlnEF kills sensitive bacteria by targeting CorC.

## Graphical abstract

Small antibacterial peptides, known as bacteriocins, have applications in food preservation and as potential therapeutics for modulating the human microbiome. Here, we identified the bacterial receptor for plantaricin EF (PlnEF), a well‐characterized two peptide bacteriocin produced by strains of *Lactobacillus plantarum,* as CorC, a putative magnesium/cobalt efflux membrane protein. The sensitivity of *L. plantarum *strains to PlnEF was magnified when strains were grown in medium containing high concentrations of MgSO_4_, CoSO_4_, CuSO_4_, or ZnSO_4_.

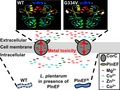

## 1 INTRODUCTION

Lactic acid bacteria (LAB) produce a diverse array of bacteriocins. Bacteriocins are ribosomally synthesized peptides with bactericidal activity and are frequently most active against species that are highly related to the producer strains (Chikindas, Weeks, Drider, Chistyakov, & Dicks, 2018). Bacteriocins, and LAB bacteriocins in particular, have received considerable interest for their potential use in food preservation and pathogen inhibition (Cotter, Hill, & Ross, 2005). The most well‐known among these bacteriocins is nisin, a class I bacteriocin produced by *Lactococcus lactis* which has been approved as a food additive in both Europe and the US since the 1980s (Gharsallaoui, Oulahal, Joly, & Degraeve, 2016). More recently, numerous LAB bacteriocin biosynthetic genes were found in human gastrointestinal tract and vaginal microbiomes, extending the potential functions of bacteriocins beyond foods to human health (Walsh et al., 2015; Zheng, Gänzle, Lin, Ruan, & Sun, 2015). However, the specific host cell targets for many of these peptides have yet to be identified, thereby limiting the expansion of bacteriocin use and accurate predictions of antimicrobial activity.

Although some bacteriocins impair intracellular components of bacteria (Acedo, Chiorean, Vederas, & van Belkum, 2018), LAB bacteriocins typically target the cell surface to cause permeabilization and cell death (Perez, Zendo, & Sonomoto, 2014). Other broad spectrum bacteriocins, such as nisin, are indiscriminate in bacteriocidal activity and bind to cell membrane‐associated components shared across taxa. For example, nisin binds to lipid II, the final precursor in peptidoglycan synthesis (Breukink et al., 1999). Bacteriocins with a narrow inhibitory spectrum are known to affect species‐ or genus‐specific proteins (Kjos et al., 2014). Initial success at identification of such receptors employed comparative genomics of bacteriocin‐sensitive and spontaneous, bacteriocin‐resistant LAB strains (Kjos et al., 2014). In this way, the receptor for the pediocin‐like bacteriocin leucocin A (targeting a mannose‐specific phosphotransferase) (Ramnath, Beukes, Tamura, & Hastings, 2000), the leaderless bacteriocin enterocin K1 (targeting a stress response membrane‐bound Zn‐dependent protease) (Ovchinnikov et al., 2017), the two‐peptide bacteriocins lactococcin G (targeting undecaprenyl pyrophosphate phosphatase) (Kjos et al., 2014), and plantaricin JK (targeting an uncharacterized protein in the amino acid‐polyamine‐organocation APC transporter protein family) were identified (Ekblad, Nissen‐Meyer, & Kristensen, 2017; Oppegård, Kjos, Veening, Nissen‐Meyer, & Kristensen, 2016).

*Lactobacillus plantarum* is a well‐characterized LAB species and most strains produce several bacteriocins called plantaricins (Nissen‐Meyer, Oppegård, Rogne, Haugen, & Kristiansen, 2010). Plantaricin EF (PlnEF) was one of the first plantaricins to be isolated and characterized for its inhibitory spectrum against LAB (Anderssen, Diep, Nes, Eijsink, & Nissen‐Meyer, 1998). It is a member of the IIb class of bacteriocins, or bacteriocins that require two different peptides in equal quantities for full activity (Nissen‐Meyer et al., 2010). PlnEF consists of the 33 residue PlnE peptide and the 34 residue PlnF peptide. PlnE has a pair of amphiphilic α‐helices at both the N‐ and C‐terminal ends (Fimland, Rogne, Fimland, Nissen‐Meyer, & Kristiansen, 2008). PlnF has a single, central α‐helix that is polar at the N‐terminal end and amphiphilic at the C‐terminus (Fimland et al., 2008). PlnE contains two GXXXG motifs characteristic of class IIb bacteriocins, while PlnF contains a GXXXG‐like motif (SXXXG). Nuclear magnetic resonance analysis of PlnEF suggests these regions provide an interaction point between the peptides (Fimland et al., 2008). Assessment of the molecular orientation that these peptides adopt in artificial, cellular membrane‐mimicking micelles revealed the most likely conformation is an anti‐parallel coupling with the N‐terminus of PlnE pointed into the cellular membrane and the N‐terminus of PlnF closer to the extracellular environment (Kyriakou, Ekblad, Kristiansen, & Kaznessis, 2016).

In this study, we employed a forward genetics approach using genome comparisons to identify the PlnEF receptor in the sensitive strain *L. plantarum* NCIMB 700965 (LP965). This strain has been used as an indicator for plantaricin biosynthesis (Kjos, Snipen, Salehian, Nes, & Diep, 2010; Moll et al., 1999) and lacks a functional PlnEF‐immunity protein Plantaricin I. Isolation and characterization of PlnEF‐resistant mutants of LP965 led to the identification of CorC*,* a putative magnesium/cobalt efflux protein, as a target of PlnEF.

## 2 RESULTS

### 2.1 Selection of PlnEF‐resistant LP965 mutants

Plantaricin E (PlnE) and plantaricin F (PlnF) were synthesized and combined in equal molar ratios (PlnEF) prior to measuring for their inhibitory activity against LP965. The MIC_50_ of PlnEF against LP965 was 12.5 nM, and LP965 did not grow in the presence of PlnEF at concentrations above 50 nM when measured over a 24 hr period (data not shown).

**Figure 1:**
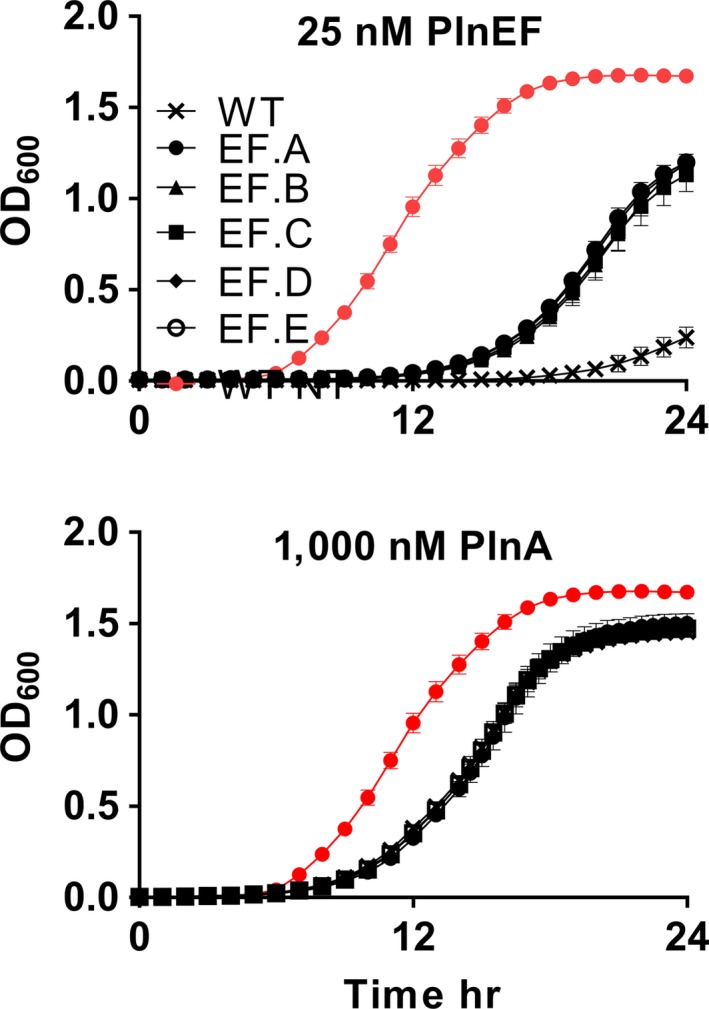
Spontaneous plantaricin EF (PlnEF) resistant mutants of LP965. *Lactobacillus plantarum* NCIMB 700965 (WT) and PlnEF‐resistant isolates were grown in MRS in the presence of 25 nM PlnEF or 1,000 nM PlnA. Red circles indicate LP965 growth in MRS without PlnEF. The avg ± *SD *of *n* = 3 replicates is shown

Spontaneous, PlnEF‐resistant mutants of LP965 were enriched by exposing the strain to 15X‐70X the MIC_50_ (187–875 nM) of PlnEF. A total of five putative PlnEF‐resistant isolates were randomly selected and confirmed for PlnEF resistance using soft agar inhibition (data not shown) and growth assays **(**Figure 1). All five isolates (designated EF.A to EF.E) exhibited a fourfold increase in PlnEF MIC_50_ values (MIC_50 _>50 nM) compared to wild‐type LP965 (WT). Growth rates and maximum optical density (as measured by OD_600_) of all isolates were increased relative to the WT strain in the presence of 25 nM PlnEF (Figure 1). Conversely, the growth rates of the PlnEF‐resistant mutants did not significantly deviate from WT LP965 when incubated in MRS lacking PlnEF (ANCOVA of growth rates *F*(3, 148) = 0.06, *p* = 0.98). The final optical density (as measured by OD_600_) and growth rates (ANCOVA, *F*(3, 44) = 1.75, *p* = 0.17) of all strains was equally reduced in the presence of 1,000 nM of the unrelated bacteriocin plantaricin A (PlnA) (Figure 1) when compared to growth without PlnA, thereby showing that PlnEF resistance was specific and not the result of changes in general, stress‐related responses to antibacterial peptides.

### 2.2 Effect of PlnEF on ATP concentrations

**Figure 2:**
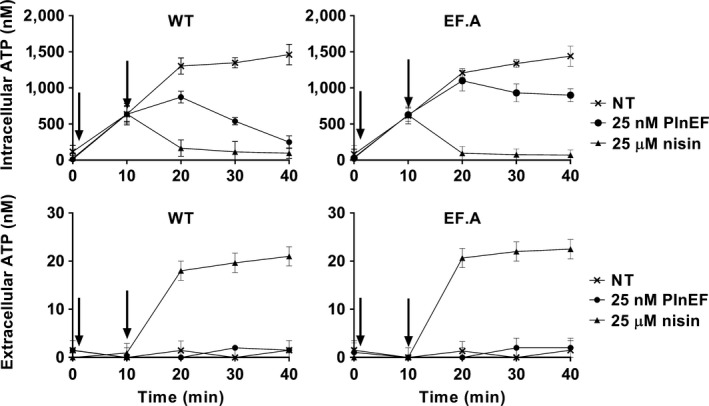
Effects of plantaricin EF (PlnEF) on cellular concentrations of ATP. Cells of LP965 and the PlnEF‐resistant isolate EF.A were initially energized with 10 mM glucose (arrow at 1 min). At 10 min (second arrow), 25 nM of PlnEF, 25 μM nisin, or water (NT) was added to each culture. The avg ± *SD* of *n* = 3 replicates is shown

**Figure A1:**
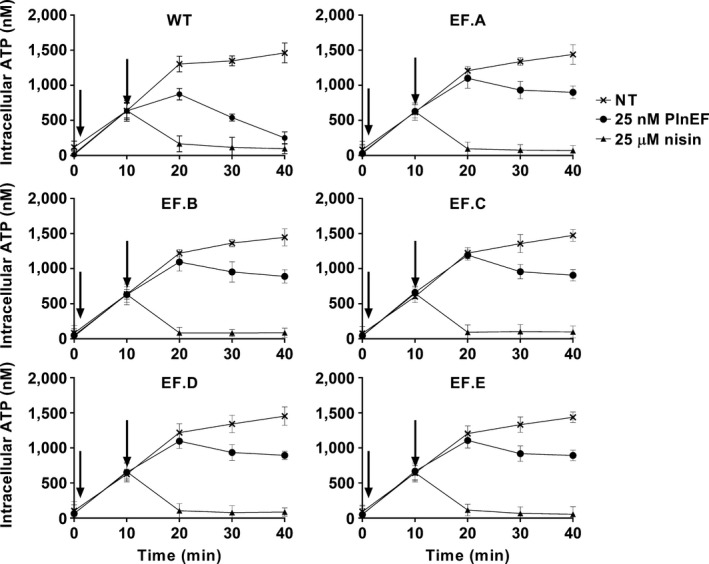
Effects of plantaricin EF (PlnEF) on intracellular concentrations of ATP. Cells of WT and PlnEF‐resistant isolates were initially energized with 10 mM glucose (arrow at 1 min). At 10 min (second arrow), 25 nM of PlnEF, 25 µM nisin, or water (NT) was added to each culture. The avg ± *SD* of *n* = 3 replicates is shown

**Figure A2:**
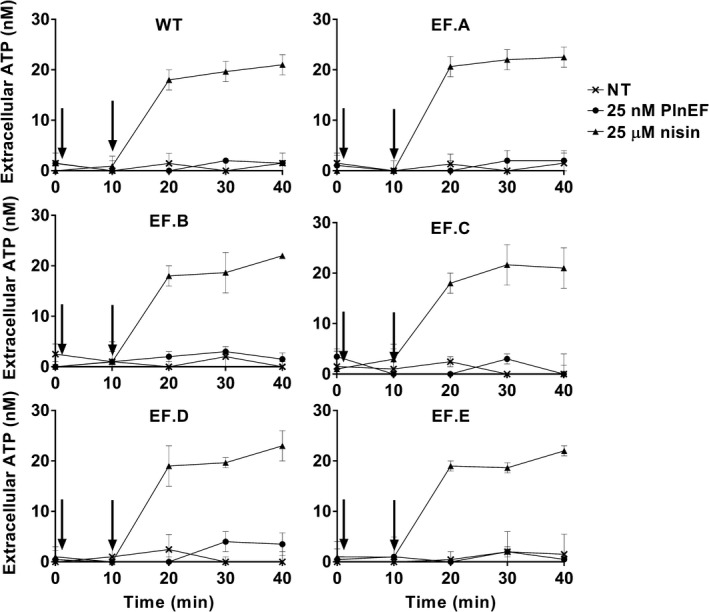
Effects of plantaricin EF (PlnEF) on extracellular concentrations of ATP. WT and PlnEF‐resistant isolates of LP965 were initially energized with 10 mM glucose (arrow at 1 min). At 10 min (second arrow), 25 nM of PlnEF, 25 µM nisin, or water (NT) was added to each culture. The avg ± *SD* of *n* = 3 replicates is shown

Because bacteriocins can disrupt energy metabolism in sensitive cells, intracellular ATP levels were measured to verify PlnEF resistance among the LP965 mutants. In the absence of PlnEF, incubation in 10 mM glucose resulted in increased intracellular ATP levels of all strains by five‐fold within 10 min (Figures 2 and A1). As expected, intracellular ATP concentrations of the WT strain declined upon exposure to 25 nM PlnEF (Figure 2), confirming the negative impact of PlnEF on cell viability. Conversely, intracellular ATP quantities of strains EF.A to EF.E increased until 20 min incubation (Figures 2 and A1), and only slightly declined thereafter. When compared to WT LP965, ATP levels of EF‐resistant strains were approximately 4‐fold higher after 40 min (*p* < 0.0001, Figures 2 and A1). The effect of PlnEF was specific to intracellular ATP quantities (Figures 2 and A2). There was no change in extracellular ATP concentrations after PlnEF challenge; whereas, all strains rapidly released ATP (approximately 20 nM) when challenged with a growth‐inhibiting concentration (25 µM) of nisin (*p* < 0.0001, Figures 2 and A2).

### 2.3 PlnEF‐resistant isolates contain a mutation in CorC, a membrane‐bound, magnesium/cobalt efflux protein

A high‐confidence reference genome for LP965 was constructed using long (PacBio) and short (Illumina) read sequence data. The DNA sequence of the PlnEF‐resistant strain EF.A was also obtained (PacBio). Alignments between the LP965 and EF.A genomes revealed three chromosomal point mutations and five gaps (a result of putative indels). To rule out sequencing errors, the eight genomic regions with sequence variations were amplified by PCR and subjected to DNA sequencing (Table A1). Only one of the eight putative mutations was verified and this mutation was found to be localized in *corC, *a gene annotated as a putative membrane‐bound, magnesium/cobalt efflux protein*. *Compared to WT LP965, a guanine was changed to a thymine in EF.A at position 846,241. This single nucleotide change results in the substitution of a valine (V) instead of a glycine (G) at amino acid residue 334 (G334V) of CorC. The *corC *gene and 100 bp flanking DNA from the four other PlnEF‐resistant isolates (EF.B–EF.E) was amplified and sequenced. This showed that all five PlnEF‐resistant mutants shared the same *corC *point mutation (translated to G334V). The expression levels of *corC *were identical between WT LP965 and the PlnEF‐resistant isolates (*p* = 0.839), indicating the point mutation was the only factor mediating resistance to PlnEF.

### 2.4 EF.A CorC G334V mutation resides in a cysteine‐β‐synthase domain

**Figure A3:**
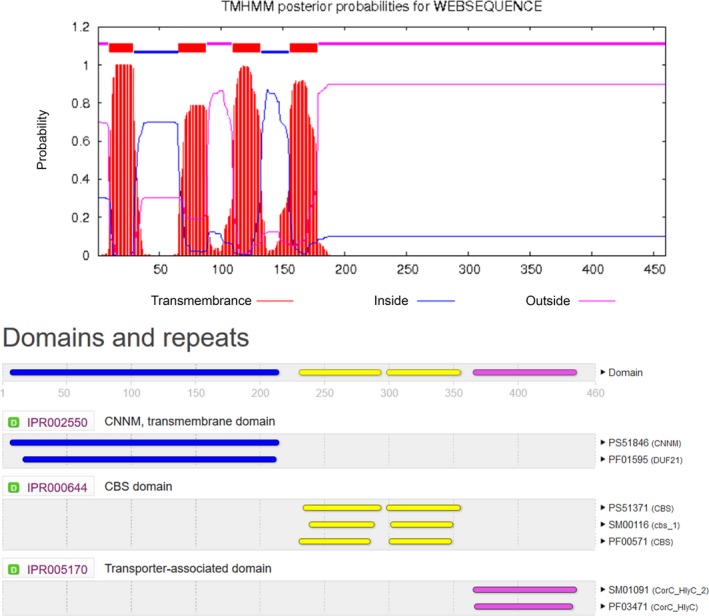
LP965 CorC domain annotation. LP965 CorC amino acid sequnce was submitted to the TMHMM server (top) and the InterPro domain annotation server (bottom). The alphanumeric strings in purple are the InterPro domain identifiers. The alphanumeric strings in black are the domain identifier links to other databases

The CorC protein is predicted to be a membrane‐bound protein containing four transmembrane domains (Figure A3). Protein annotation predicts two cysteine‐β‐synthase domains (CBS, also known as Bateman domains (Baykov, Tuominen, & Lahti, 2011)) and two transporter‐associated domains (PFAM03471, Figure A3). The CorC G334V mutation in strains EF.A–EF.E is localized in the second of the two CBS domains.

**Figure 3:**
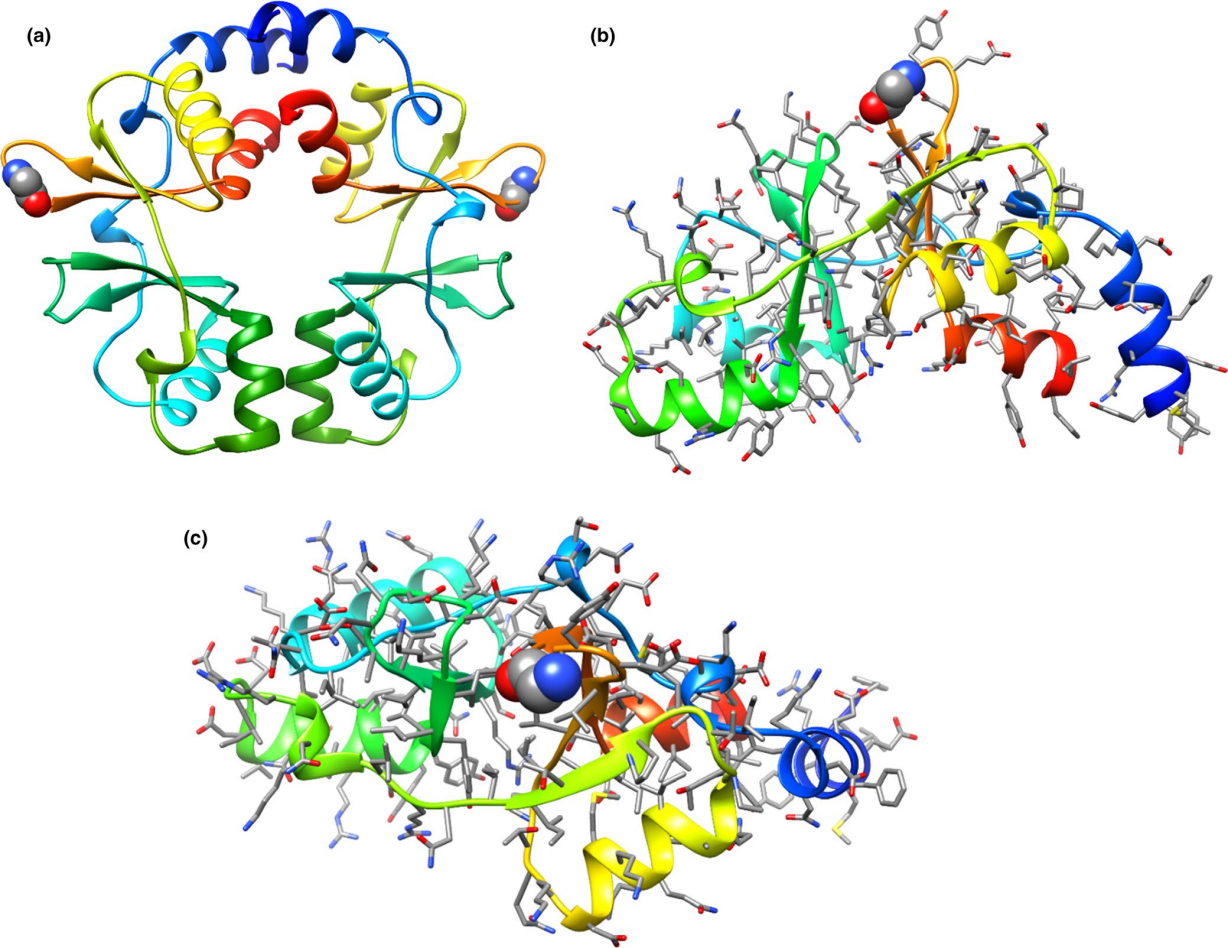
Structural model of the LP965 CorC protein. (a) Top‐down view of proposed homodimer formed by tandem CBS domains. (b) Top‐down and (c) side‐views of (monomer) LP965 CorC protein region from Y216 to G351 shown in ribbon representation and colored by a rainbow scheme from N‐terminus region (blue) to C‐terminus region (red). All sidechains are shown in stick representation, except for G334, which is shown in space‐filling representation. Template‐based modeling was accomplished using PDB: 3OCO

**Figure A4:**
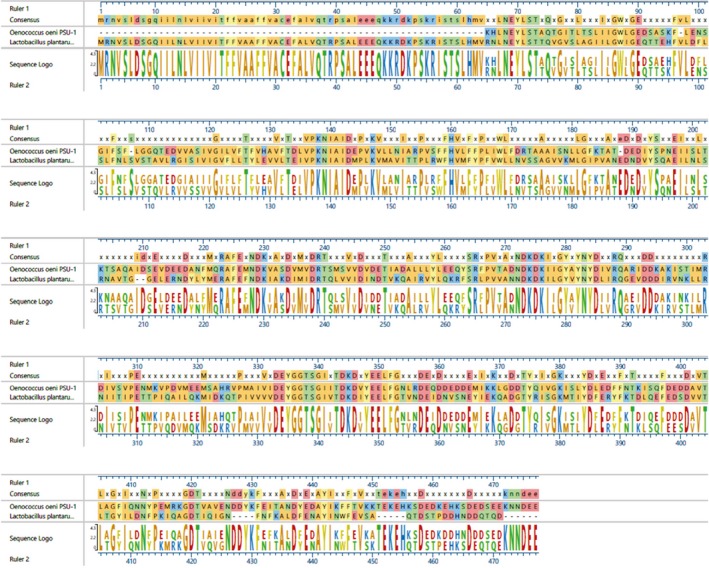
Alignment of CorC amino acid sequences. Amino acid sequences from LP965 and *Oenococcus oeni* were aligned using MUSCLE. Due to gaps in the alignment, the mutated glycine residue in LP965 (334) in this figure is in position 336

To elucidate a potential molecular basis for CorC interactions with PlnEF, structural modeling was performed for the WT LP965 CorC protein sequence region from Y216 to G351, encompassing the C‐terminal CBS. This was accomplished using the resolved crystal structure of the CorC protein produced by *Oenococcus oeni* (PDB ID:3OCO), which shares 41% amino acid identity to CorC in LP965 (Figure A4). Our Y216 to G351 model of LP965 CorC indicated that this region might be involved in a dimerization interface (Figure 3a), a result that is consistent with the model of 3OCO. The template‐based model also revealed that the G334V CorC mutation in PlnEF‐resistant strains of LP965 resides in a loop region between two β‐strands (Figure 3b,c).

### 2.5 PlnEF increases the effects of metal stress

**Figure A5:**
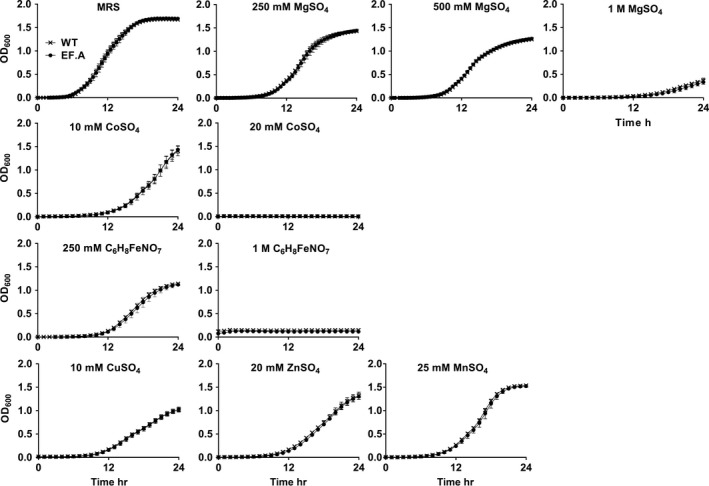
WT and EF.A metal sensitivity. WT and EF.A LP965 were grown in MRS or MRS supplemented with the indicated growth‐inhibiting concentration of metal salt

Because CorC is annotated as a putative magnesium/cobalt efflux protein, we sought to characterize whether the CorC G334V mutation altered LP965 sensitivity to different divalent metal cations. However, there were no significant differences in the growth rates or final optical densities between LP965 and the CorC mutant when incubated in MRS in the presence of growth‐inhibiting concentrations of MgSO_4_, CoSO_4_, C_6_H_8_FeNO_7_, CuSO_4_, ZnSO_4_, or MnSO_4_ (Figure A5).

**Figure 4:**
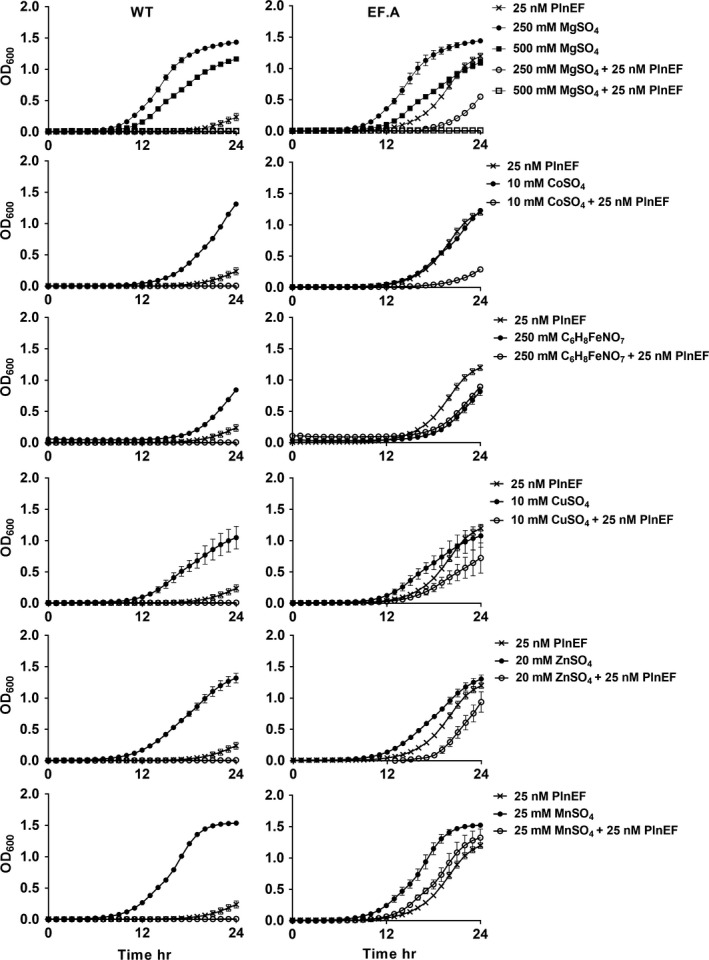
Additive effects of plantaricin EF (PlnEF) and metals on *Lactobacillus plantarum* growth. WT LP965 and the EF‐resistant strain EF.A were incubated in MRS supplemented with 25 nM PlnEF or MRS with 25 nM PlnEF and the indicated metal salt. The avg ± *SD* of *n* = 3 replicates is shown

Next, we investigated whether the CorC mutation altered *L. plantarum *growth in the presence of both PlnEF and high concentrations of metal cations. Growth of WT LP965 was completely inhibited in MRS containing additional MgSO_4_, CoSO_4_, C_6_H_8_FeNO_7_, CuSO_4_, ZnSO_4_, or MnSO_4_ and 25 nM of the PlnEF bacteriocin (Figure 4). Growth of EF.A was not affected by the inclusion of either C_6_H_8_FeNO_7_ or MnSO_4 _in MRS with 25 nM PlnEF (Figure 4). Surprisingly, however, MRS containing supplemental MgSO_4_, CoSO_4_, CuSO_4_, or ZnSO_4_, also resulted in significant impairments to the growth of the EF.A mutant when PlnEF was present (Figure 4). A dose dependency was found such that strain EF.A initiated growth by 20 hr in the presence of supplemental 250 mM MgSO_4_, but not 500 mM MgSO_4_ (Figure 4). These results show that PlnEF acts synergistically with metals to inhibit the growth of LP965 and that the G334V mutation is not sufficient to prevent cell damage at high concentrations of MgSO_4_, CoSO_4_, CuSO_4_, and ZnSO_4_.

### 2.6 Heterologous expression of wild‐type LP965 *corC* increases *L. casei* BL23 sensitivity to PlnEF

Numerous attempts to truncate or delete LP965 *corC *were unsuccessful using methods commonly applied for genetic modification of *L. plantarum *(Aukrust, Brurberg, & Nes, 1995). Efforts to delete or truncate *corC *in *L. plantarum *NCIMB 8,826 (LP8826) were also unsuccessful. Because LP8826 is amenable to genetic manipulation (Yin et al., 2017), we concluded that CorC is likely an essential protein for *L. plantarum*.

**Figure 5:**
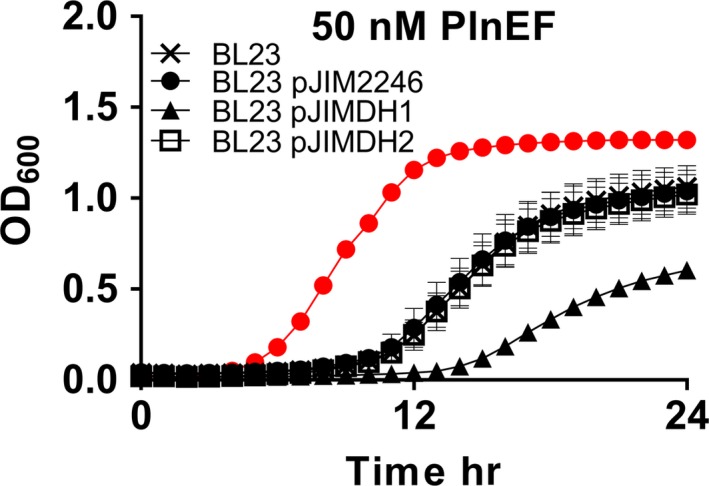
Heterologous expression of LP956 and EF.A CorC in *L. casei* BL23. L. casei was grown in MRS with 50 nM of plantaricin EF (PlnEF). pJIMDH1 contains corC from WT and pJIMDH2 contains CorC from strain EF.A. Red circles indicate BL23 growth in MRS without PlnEF. The avg ± *SD* of *n* = 3 replicates is shown

Therefore, to confirm that the G334V CorC mutation is required for PlnEF resistance, we introduced *corC *from WT LP965 and the EF.A mutant into *L. casei *strain BL23. Strain BL23 contains a homolog to CorC (59% amino acid identity), but is at least twice as resistant to PlnEF as LP965 (MIC_50 _= 25 nM). Expression of wild‐type LP965 *corC *in *L. casei *BL23 increased the sensitivity of that organism to PlnEF from 25 nM to 12.5 nM (Figure 5). Conversely, no increase in PlnEF sensitivity was found when the EF.A CorC G334V mutant was expressed (MIC_50_ = 25 nM) (Figure 5). The growth rates (ANCOVA, *F*(2, 134) = 0.03, *p* = 0.90) and final OD_600_ of BL23 or BL23 harboring either *corC *variant were equivalent in MRS lacking PlnEF. The expression levels of LP965 and EF.A *corC *in *L. casei *were also equivalent (*p* = 0.745). These results confirm that wild‐type LP965 *corC* increases sensitivity to PlnEF and that the single G334V amino acid substitution is sufficient to confer resistance.

## 3 DISCUSSION

We identified CorC, a putative magnesium/cobalt exporter, as the receptor for the *L. plantarum *bacteriocin PlnEF. These findings are in agreement with previous studies on PlnEF showing that this bacteriocin causes cation efflux (Moll et al., 1999). We hypothesize that PlnEF anchors to CorC and either inserts into the lipid bilayer directly or, alternatively, PlnEF inserts through CorC to cause disrupted metal homeostasis. In either case, the activity of PlnEF is distinct from the *L. plantarum *bacteriocin Plantaricin JK which is known to induce anion efflux and for which the cell surface receptor was identified as a protein in the APC transporter family (Oppegård et al., 2016). The results are similarly consistent with prior work showing the bacterial receptor for PlnEF is different from lactococcin A and other class II pediocin‐like bacteriocins known to bind to proteins in the mannose phosphotransferase system (Diep, Skaugen, Salehian, Holo, & Nes, 2007). Our findings also conform with the expectation that there is a specific protein receptor for PlnEF which causes targeted damage to the cell, as opposed to receptors for broad‐spectrum bacteriocins like nisin which cause more pervasive effects on the cell membrane and the release of intracellular ATP (Breukink et al., 1999).

Because metal concentrations vary significantly throughout microenvironments, bacteria contain dedicated proteins for the import of essential metals and export of excess/toxic metals (Barwinska‐Sendra & Waldron, 2017). The *L. plantarum *reference strain WCFS1 contains 42 annotated metal cation transporters (Kleerebezem et al., 2003). *L. plantarum *strains have long been known to have a relatively high requirement for manganese (MacLeod & Snell, 1947), and the best understood metal transport systems in this species are those with affinity for Mn^2+^ (Groot et al., 2005). Presently, transport systems for magnesium and the trace metals Zn^2+^, Co^2+^, Cu^2+ ^and Fe^2+^ have not been well characterized in *L. plantarum*. In other bacteria, magnesium import, in particular, has been extensively studied and linked to four classes of transporters (Groisman et al., 2013; Moomaw & Maguire, 2008; Shin et al., 2014). CorA, the most widely distributed of these transporters, has been shown to maintain both Mg^2+ ^and Co^2+ ^homeostasis in *L. lactis* (Mills et al., 2005). Some bacteria also carry systems for Mg^2+^ export, although this function is not as well understood. Putative Mg^2+^ efflux proteins have been identified as CorB, CorC, and CorD in *Salmonella* (Gibson, Bagga, Miller, & Maguire, 1991) and *Shigella* (Zhang, Ren, Zhu, Li, & Wang, 2010) and YhdP in *Bacillus *(Akanuma et al., 2014). These proteins might also export other metals such as Zn^2+^, as indicated for CorB and CorC in *Pseudomonas stutzeri* (Vaccaro et al., 2016).

CBS domains are frequently found in two to four tandem copies in both cytosolic‐ and membrane‐associated proteins and are present in proteins from all domains of life (Baykov et al., 2011). In eukaryotes, the cyclin M (CNNM) family of proteins mediate Mg^2+^ transport and share some structural similarities to bacterial CorC and CorB including the C‐terminal CBS pairs (Hirata, Funato, Takano, & Miki, 2014). The CBS domains of magnesium transporters are reported to be important for magnesium and ATP binding (Armitano, Redder, Guimarães, & Linder, 2016; Baykov et al., 2011). Recently, a *Staphylococcus aureus* protein with a domain structure similar to *Salmonella *CorB (magnesium protection factor A (MpfA); SA00657) was found to be essential for *S. aureus *growth in magnesium concentrations as low as 10 mM (Armitano et al., 2016). Point mutations in a conserved glycine residue at position 326 in an intracellular CBS domain of MpfA resulted in increased Mg^2+^ sensitivity. The LP965 CorC G334V mutation is also in a CBS domain, however, because the growth rates of the CorC mutant EF.A and WT LP965 were equivalent in MRS with high concentrations of MgSO_4_, our data suggest that the glycine residue at position 334 does not participate in metal binding. Instead of binding metals, the G334V mutation could result in steric inhibition altering the target site of PlnEF. Therefore it was notable the EF.A CorC mutation was not sufficient to sustain resistance to PlnEF in the presence of high levels of either MgSO_4_ or the trace metals CoSO_4_, CuSO_4_, and ZnSO_4_. This increase in PlnEF sensitivity could be due to changes in CorC conformation induced by external metal concentrations which result in increased bacteriocin binding. Such a possibility is supported by experiments visualizing CorC from *Shigella flexneri* altering conformations in response to ATP binding (Zhang et al., 2010). The magnesium import protein CorA has also been shown to change conformational states in response to magnesium binding (Matthies et al., 2016), thereby indicating a dynamic process.

PlnEF‐resistant mutants were only incrementally (4X) more resistant to the bacteriocin than WT LP965. By comparison, lactococcin G‐resistant mutants of *L. lactis *had MIC_50_ values at least 1,000 to 10,000 times greater than the wild‐type strain (Kjos et al., 2014). This difference could be due to the fact that those mutations were predicted to encode truncated forms of the Upp receptor protein (Kjos et al., 2014); whereas only a single amino acid substitution in CorC was found among the five LP965 PlnEF‐resistant mutants examined here. Heterologous expression of the LP965 and EF.A CorC proteins in *L. casei *also confirmed the importance of the single G334V point mutation in conferring PlnEF resistance. Because we did not find other mutants and because we were unable to construct a CorC deletion mutant in either LP965 or LP8826, our results indicate that similar to *S. aureus *MpfA (Armitano et al., 2016), CorC is essential for maintaining magnesium metal homeostasis in *L. plantarum*.

Determining how bacteriocins exert antimicrobial activity is important for elucidating microbe‐microbe interactions and application potential in food and intestinal ecosystems. Identification of bacteriocin cellular receptors will advance our understanding of the inhibitory spectra, functional significance, and resistance mechanisms associated with the myriad of bacteriocins currently known. We recently demonstrated that the PlnEF system is important for *L. plantarum *mediated protection against diet induced obesity in mice (Heeney et al., 2018). The gut microbiota and colonic metabolomes were not altered with *L. plantarum *consumption. Instead, the *L. plantarum* plantaricin system was correlated with increased production of the tight junction protein ZO‐1 in the intestinal epithelium. Application of the purified peptides on differentiated Caco‐2 monolayers also prevented disruptions to epithelial barrier integrity caused by proinflammatory cytokines (Heeney et al., 2018), thereby indicating direct interactions of the bacteriocin with epithelial cells. Identification of the PlnEF‐binding sites should clarify the exact mechanisms for which the bacteriocin alters metal homeostasis and supports the identification of other molecular targets for these peptides in the mammalian intestine.

## 4 EXPERIMENTAL PROCEDURES

### 4.1 Bacterial strains and growth conditions

Strains and plasmids used in this study are listed in Table 1. *Lactobacillus *strains were grown in Lactobacilli MRS (MRS, BD Biosystems) broth. LP965 strains were grown without aeration at 30°C. *L. casei *BL23 and *L. plantarum *NCIMB 8,826 (LP8826) were grown without aeration at 37°C. *Escherichia coli *DH5α was grown in Lennox lysogeny broth (Teknova) with aeration (250 rpm) at 37°C. When appropriate, 5 or 100 µg/ml chloramphenicol (Cm^R^) and 5 or 300 µg/ml erythromycin (Ery^R^) (Sigma‐Aldrich) was included in the media for *Lactobacillus *and *E. coli*, respectively. To test the effects of plantaricin A (PlnA) on *L. plantarum *growth, PlnA was added to MRS broth at a final concentration of 1,000 nM. This concentration was selected because it resulted in some inhibition, reducing the final optical density of LP965 compared to when grown in MRS without bacteriocin. To measure sensitivity to metal cations, sterile solutions of anhydrous MgSO_4_, CoSO_4_.7H_2_O, ZnSO_4_.7H_2_O, CuSO_4_.5H_2_O, MnSO_4_.H_2_O, or ferric ammonium citrate (C_6_H_8_FeNO_7_) were added to MRS to reach final supplemental concentrations of 500 mM or 1 M MgSO_4_, 10 mM or 20 mM CoSO_4_.7H_2_O, 20 mM ZnSO_4_.7H_2_O, 10 mM CuSO_4_.5H_2_O, 25 mM MnSO_4_.H_2_O, and 500 mM or 1 M C_6_H_8_FeNO_7_. The concentrations were selected to test the range of LP965 sensitivity to those metal salts.

**Table 1:** Strains and plasmids used in this study

	Description	Reference
Strains		
*L. plantarum *NCIMB 700,965	PlnEF‐sensitive strain	(Sherwood, 1939)
*L. plantarum *NCIMB 8,826	PlnEF‐producing strain, parent of WCFS1	(Hayward & Davis, 1956)
*L. casei *BL23	PlnEF‐resistant reference strain	(Maze et al., 2010)
*E. coli *DH5α	*fhuA2 lac(del)U169 phoA glnV44 Φ80' lacZ(del)M15 gyrA96 recA1 relA1 endA1 thi−1 hsdR17, *amplification of cloning vector	(Taylor, Walker, & McInnes, 1993)
Plasmids		
pJIM2246	Cm^R^, low‐copy cloning vector	(Renault et al., 1996)
pJIMDH1	pJIM2246 derivative containing strain 965 *corC*	This work
pJIMDH2	pJIM2246 derivative containing strain EF.A *corC*	This work
pRV300	Ery^R^ Amp^R^, *E. coli* Ori pMB1, integrative vector	(Leloup, Ehrlich, Zagorec, & Morel‐Deville, 1997)
pRVDH1	pRV300 derivative used for deletion of *corC*	This work

### 4.2 Bacteriocin peptide synthesis

The full‐length peptide sequences of plantaricins taken from the published genome of *L. plantarum *WCFS1 (Refseq: NC_004567.2) were downloaded from NCBI and trimmed to delete export‐signal peptide sequences. Leaderless forms of plantaricin E (FNRGGYNFGKSVRHVVDAIGSVAGIRGILKSIR), and plantaricin A (PlnA) (KSSAYSLQMGATAIKQVKKLFKKWGW) were chemically synthesized by Genscript. Plantaricin F (VFHAYSARGVRNNYKSAVGPADWVISAVRGFIHG) was synthesized by Thermo‐Fisher. Peptides were 98%–99% pure and diluted in ultra‐pure, molecular grade water (Ambion) prior to being stored at −20°C until use.

### 4.3 Bacteriocin activity assays

The antimicrobial activity of PlnEF against target cells was tested as previously described (Kjos et al., 2014) using a spectrophotometer to monitor optical density (Synergy 2, Biotek instruments). The minimum inhibitory concentration (MIC_50_) was defined as the peptide concentration (the sum of both peptides [in a 1:1 ratio]) that inhibited growth by 50% after 6 hr incubation.

### 4.4 Isolation of PlnEF‐resistant mutants

LP965 PlnEF‐resistant strains were selected as previously described (Kjos et al., 2014). Five wild‐type (WT) LP965 colonies were inoculated into separate 10 ml volumes of MRS broth containing 70X the MIC_50_ of PlnEF (875 nM) and incubated at 30°C for 72 hr. Aliquots of 100 µl from each culture were then plated onto MRS agar plates containing 15X the MIC_50_ of PlnEF (187 nM) and incubated for 48 hr. One putative resistant colony from each plate (out of a total of approximately 30 colonies across all plates) was then incubated in MRS broth for 24 hr. Overnight cultures were diluted in phosphate buffered saline (Corning) (PBS) and then 100 µl of each culture was plated onto MRS agar containing 15X PlnEF at an estimated density of 1 × 10^6^_ _CFU ml^−1^ and incubated for 24 hr. Out of a total of 420 putative resistant mutants, one PlnEF resistant colony was picked from each PlnEF‐containing MRS plate and grown in MRS broth without the bacteriocin for approximately 36 generations. PlnEF resistance was confirmed for each of the isolates prior to preparation of glycerol stocks and storage at −80°C.

### 4.5 Quantification of corC expression levels

RNA was extracted from three separate exponential‐phase cultures of WT LP965, EF.A, or *L. casei *BL23 strains grown in MRS as previously described (Tachon, Lee, & Marco, 2014). cDNA was synthesized with the RETROscript kit (Ambion) according to the manufacturer's instructions. Expression of *corC* was quantified for each strain by quantitative PCR (qPCR). Reactions were performed on an Applied Biosystems 7500 Real‐time thermocycler using 4 ng of RNA, 0.2 µM of each primer (Table A2), and Fast Sybr Green Master Mix (Thermo) under the following conditions: 10 min at 95°C and then 40 cycles of 10 s 95°C and 30 s 60°C followed by melt curve analysis. Expression was quantified by the 2^−∆∆Ct^ method using *rpoB *as an internal control and WT expression levels as the reference condition.

### 4.6 Soft‐agar growth inhibition assay

Single colonies of naïve and PlnEF‐resistant LP965 strains were used as indicator strains in soft agar assays as previously described (Kjos et al., 2014) with strain *L. plantarum *NCIMB 8826 used as the producer. A resistant LP965 phenotype was detected by a reduced zone of inhibition compared to the naïve strain after overnight incubation at 37°C.

### 4.7 Isolation of genomic DNA and whole genome sequencing

DNA was isolated from WT and EF.A stationary phase cultures grown in MRS by phenol‐chloroform extractions, followed by ethanol precipitation (Sambrook & Russell, 2006). DNA Sequencing was performed using a PacBio RSII instrument with a desired insert size of 10 kb and P6C4 chemistry according to manufacturer's instructions at the University of California, Davis, DNA Technologies Core (http://dnatech.genomecenter.ucdavis.edu).

Sequence SMRTcell files were downloaded from the UC Davis Sequencing core and imported to the PacBio SMRT portal graphical interface unit (http://www.pacb.com). The genome of LP965 was assembled first de novo using the hierarchical genome assembly protocol 2 (RS_HGAP_assembly.2) with default parameters. The final assembly resulted in an average 310X coverage (chromosome of 3,015,426 bp, and plasmids with the following sizes, plasmid 1:66,439 bp, plasmid 2:52,109 bp, plasmid 3:41,818 bp, plasmid 4:23,484 bp, and plasmid 5:16,940 bp). To ensure a high‐quality reference genome, short read archive data generated from LP965 (accession number: SRR1553345) was downloaded from NCBI (https://www.ncbi.nlm.nih.gov/sra). A total of 4.7 million 75 bp (349.3 Mb) single end Illumina reads were assembled into 307 contigs (Cellera assembler, default parameters, https://sourceforge.net/projects/wgs-assembler/). These contigs were then aligned to the PacBio assembly data (RS_AHA_Scaffolding.1) to generate a single high confidence FASTA file with one chromosome and five plasmids (LP965). The LP965 genome sequence was then uploaded to RAST (http://rast.nmpdr.org/) (Aziz et al., 2008) for annotation and further analyzed with Seqbuilder (DNASTAR).

The LP965.EF.A strain genome was assembled using the high‐quality, wildtype genome of LP965 as a reference and the program (RS_Resequencing.1) with default parameters except for the following adjustments: minimum subread length of 8 kb, minimum read quality 75, minimum polymerase read length of 12 kb. This resulted in one chromosome and five plasmids with an average coverage of 193X. To identify variants between the wild type and EF.A strain, each genome was compiled into single FASTA files and aligned with MAUVE 2.4.0 (Darling, Mau, & Perna, 2010). This resulted in five indels and three single nucleotide polymorphisms found on the chromosome.

Each region with conflicting base calls was used to design PCR primers to amplify a 250 bp segment of DNA (Table A1). PCR was conducted with Takara ex‐Taq according to manufacturer's instructions. The PCR products were purified with the Wizard SV gel and PCR Clean‐Up system (Promega) before being submitted for bi‐directional DNA sequencing at the University of California Davis Sequencing core (http://dnaseq.ucdavis.edu/).

### 4.8 Cellular ATP quantification

Intracellular and extracellular ATP levels were determined in triplicate cell cultures. Overnight cultures were washed in PBS and suspended in PBS with 10 mM glucose and either water (sham), 25 nM PlnEF or 25 µM nisin (Sigma). Nisin was added at concentrations that limited growth of LP965 to a similar extent as 25 nM PlnEF. At indicated time points, 120 µl samples were collected and centrifuged for 2 min at 20,000X g. The supernatant (extracellular ATP) was aspirated and mixed at a ratio of 1:1 with dimethyl sulfoxide (DMSO, Sigma). The cell pellet (intracellular ATP) was then mixed with 100 µl DMSO. ATP concentrations were determined with the Invitrogen ATP Quantification kit according to manufacturer's instructions and luminescence was quantified according to a standard curve on a Biotek Synergy 2 spectrophotometer.

### 4.9 CorC mutagenesis

LP965 was not amenable to genetic manipulation with techniques described to transform this strain previously (Aukrust et al., 1995) or by adjusting parameters (glycine concentrations, recovery times, voltage) described therein. Repeated attempts to delete the *corC *open reading frame (ORF) in LP8826 (WCFS1 lp_2671) using methods previously established for clean deletions in this strain (Yin et al., 2017) or variations of those methods (glycine concentrations, recovery times, voltage, incubation temperature) were equally unsuccessful. Primers used for these attempts are listed in Table A3.

### 4.10 Heterologous expression of *L. plantarum* corC in *L. casei* BL23

The *corC *genes from LP965 and EF.A were amplified with primers A and D using genomic DNA as a template (Table A3). The product was digested with EcoR1‐HF and SacI‐HF (New England Biolabs) and ligated into the multiple cloning site of pJIM2246 (Renault, Corthier, Goupil, Delorme, & Ehrlich, 1996) resulting in pJIMDH1 (WT LP965 CorC) and pJIMDH2 (EF.A CorC). *E. coli *DH*5*α was transformed and selected for as previously described (Yin et al., 2017). Plasmids were isolated from *E. coli *and amplified by PCR using *corC *primers (Table A1) and DNA sequencing for verification. *L. casei *BL23 electrocompetent cells were prepared and transformed as previously described (Welker, Hughes, Steele, & Broadbent, 2015). Briefly, freshly prepared BL23 cells were electroporated with 400 ohms, 2 kV, and 25 μF and then immediately transferred to MRS supplemented with 0.5 M sucrose. The cells were then incubated for 4 hr and transformants were selected by plating serial dilutions on selective medium. PCR and DNA sequencing confirmed the presence of the plasmids in *L. casei *BL23.

### 4.11 Structural modeling of CorC protein

Co‐evolutionary constraints were determined using the GREMLIN server (http://gremlin.bakerlab.org/) (Kamisetty, Ovchinnikov, & Baker, 2013). A 3D structure model of the CorC protein from LP965 was performed using relax application (Conway, Tyka, DiMaio, Konerding, & Baker, 2014; Khatib et al., 2011; Nivón, Moretti, & Baker, 2013; Tyka et al., 2011) in Rosetta structural modeling software (Alford et al., 2017; Bender et al., 2016; Rohl, Strauss, Misura, & Baker, 2004) and the x‐ray structure of a CorC‐like protein from of *Oenococcus oeni* PSU (PDB ID: 3OCO) as a template. Sequences were aligned with MUSCLE (Edgar, 2004) and % identity between the CorC protein sequence and 3OCO was 41% (Figure A4). All structural modeling figures were generated using the UCSF Chimera package (Pettersen et al., 2004). The highest ranked Rosetta model of CorC protein was used to investigate the mutational hotspot region. Further domain annotation was accomplished by submitting the LP965 CorC protein sequence to the TMHMM server (Krogh, Larsson, Heijne, & Sonnhammer, 2001) and the InterPro online server http://www.ebi.ac.uk/interpro/(Finn et al., 2017).

### 4.12 Statistics

Growth assays were conducted with technical replicates in triplicate and data are representative of at least two independent experiments. Data are presented as average values ± *SD*. Analysis of covariance (ANCOVA) was used to determine whether the growth rates of cultures in logarithmic phase were statistically different from one‐another. Student's *t *test with Welch's correction was used to determine significant differences between final optical densities of cultures and levels of *corC* expression.

## CONFLICT OF INTERESTS

The authors declare that the research was conducted in the absence of any commercial or financial relationships that could be construed as a potential conflict of interest.

## AUTHORS CONTRIBUTION

DDH wrote the manuscript and designed and conducted experiments. VYY produced 3D models and edited. MLM designed experiments and edited the manuscript.

## ETHICS STATEMENT

None required.

## Data Availability

LP965 WT sequence data was deposited into the National Center for Biotechnology Information (https://www.ncbi.nlm.nih.gov/) with accession numbers CP023490 to CP023495. The EF.A PlnEF‐resistant strain was deposited in NCBI with accession numbers CP026505 to CP026510.

## APPENDIX 1

**Table A1:** Primers used for DNA sequencing

Primers	DNA Sequence	Description of gene	Putative mutation location in CP023490	Reference
*lipP*For	CAAAAATACCGGAGGCGTAA	FIG00745599, lipoprotein precursor	SNP: G1,960,045A	This study
*lipP*Rev	ATTGCGACGCCTCATAATTC			
*corC*For	CCCGCTGACTACGATAGACC	putative magnesium efflux protein	SNP: G846,241T	This study
*corC*Rev	TGCCATTTTAAGTAGTGCGTGT			
*topo*For	CCGGCAATACTTTGTTCGAT	DNA topoisomerase I	SNP: G119,959A	This study
*topo*Rev	GAAATTGCGGACCAGGTCTA			
*oppA*For	CAACATAGCCGGTCCAGTTT	Oligopeptide ABC transporter	Gap: A del 1,596,580	This study
*oppA*Rev	CGTCGGCCTTATTAGGATCA			
*abcT*For	TGGTTCAAATGGTGCTGGTA	ABC transporter ATP‐binding protein	Gap: in G 2,679,932	This study
*abcT*Rev	TTGGGCTTTTTCCTCATCAC			
*mutL*For	CCTTGAATGGCAACCATCTT	DNA mismatch repair protein	Gap: del G 550,025	This study
*mutL*Rev	TTGATGACGCTGAAGTCCTG			
*carB*For	CCGGCAATACTTTGTTCGAT	Carbamoyl‐phosphate synthase large chain	Gap: del C 880,235	This study
*carB*Rev	GAAATTGCGGACCAGGTCTA			
*pts*PFor	TCGTGTCCTTCGCCTTAACT	Promoter region of phosphotransferase system	Gap: del T 2,558,123	This study
*ptsP*Rev	AACTGGTGCCCAGATTTGAC			

**Table A2:** Primers used for qPCR

Primers	DNA sequence	Description of gene	Reference
*corC*For	GCCAAGGTGAAGTTGACGAT	putative magnesium efflux protein	This study
*corC*Rev	CCACTGGTCCCACCATATTC		
Lplant_*rpoB*For	GGCAGAACAGATCAAGGAAGG	RNA‐directed polymerase subunit B	(Marco, Bongers, Vos, & Kleerebezem, 2007)
Lplant_*rpoB*Rev	TATCCACTTCGGCACGCTTA		
Lcasei_*rpoB*For	GACCTTGCGTTTAACCAACC	RNA‐directed polymerase subunit B	This study
Lcasei_*rpoB*Rev	AACGGACCAACTGAGAAACG		

**Table A3:** Primers used for cloning

Primer	Sequence^a^	Description of gene region	Plasmid construction	Reference
EcoR1‐*corC*‐For (A)	CCGGAATTCAGCACCATTCATTGGAAACG	Upstream of *corC. * Restriction site	pJIMDH1, pJIMDH2, pRVDH1	This study
CorC_up_rev_linker (B)	*GAAGCAGCTCCAGCCTACAC*CGGTTGAGACGGATAGGTTG	Upstream of *corC. linker sequence*	pRVDH1	This work
CorC_down_for_linker (C)	*GTGTAGGCTGGAGCTGCTTC*CCGATGTTGTCACTTTGACC	Downstream of *corC. linker sequence*	pRVDH1	This study
SacI‐*corC*‐Rev (D)	GGGGAGCTCTCCCTTAAATCAACCGCATC	Downstream of *corC.* Restriction site	pJIMDH1, pJIMDH2, pRVDH1	This study

a Restriction enzyme sites are underlined; linker DNA sequences are italicized.
